# Cue properties change timing strategies in group movement synchronisation

**DOI:** 10.1038/srep19439

**Published:** 2016-01-19

**Authors:** Juliane J. Honisch, Mark T. Elliott, Nori Jacoby, Alan M. Wing

**Affiliations:** 1 School of Psychology and Clinical Language Sciences, University of Reading, Reading, RG6 6AL, UK; 2 Institute of Digital Healthcare, University of Warwick, Coventry, CV4 7AL, UK; 3 Department of Brain and Cognitive Sciences, Massachusetts Institute of Technology, Cambridge, Massachusetts, USA; 4 Music Department, Bar Ilan University, Ramar Gan, Israel; 5 School of Psychology, University of Birmingham, Edgbaston, B15 2TT, UK

## Abstract

To maintain synchrony in group activities, each individual within the group must continuously correct their movements to remain in time with the temporal cues available. Cues might originate from one or more members of the group. Current research suggests that when synchronising movements, individuals optimise their performance in terms of minimising variability of timing errors (asynchronies) between external cues and their own movements. However, the cost of this is an increase in the timing variability of their own movements. Here we investigate whether an individual’s timing strategy changes according to the task, in a group scenario. To investigate this, we employed a novel paradigm that positioned six individuals to form two chains with common origin and termination on the circumference of a circle. We found that participants with access to timing cues from only one other member used a strategy to minimise their asynchrony variance. In contrast, the participant at the common termination of the two chains, who was required to integrate timing cues from two members, used a strategy that minimised movement variability. We conclude that humans are able to flexibly switch timekeeping strategies to maintain task demands and thus optimise the temporal performance of their movements.

For social groups, moving together in time is a common biological phenomenon that exists in humans^1^,^2^ as well as in non-human animals^3^,^4^,^5^. In humans, such coordinated actions are common in group activities, for example music^6^,^7^, dance^8^,^9^, and rowing^10^. Moreover, coordinated movements can emerge spontaneously within group social interactions^11^,^12^.

Humans are able to extract and reproduce intervals from a rhythmic beat with relative ease^13^, usually after observing just 2–3 beats. However, maintaining *synchrony* with a rhythmic cue relies on precise timekeeping to ensure movements remain in phase with the beat. Such accuracy is achieved through continuous correction of the timing errors (asynchronies) between one’s own movements and the rhythmic cues^14^,^15^. The error correction process can be described by a linear phase correction model, with the timing of each movement adjusted by a proportion (denoted as the correction gain, 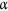) of the last asynchrony between movement and cue onset^15^,^16^. This model has been shown to describe the error correction process in both upper^17^,^18^,^19^ and lower limb movements^20^,^21^,^22^,^23^ suggesting there is a common timing correction mechanism regardless of effector^23^,^24^.

The modality, and more generally the reliability, of the cue dictate the proportion of the correction to the last asynchrony. Cues less reliable in terms of increased variance of the estimated onset times result in lower correction gains^23^. Similarly, lower reliability in the proprioceptive feedback of one’s own movement timing also results in observation of lower correction gains^17^.

Inevitably, increased variability in the cue leads to increased variability in the asynchronies. It is claimed that when synchronising movements with a rhythmic cue, a strategy of minimising the asynchrony variability is used to achieve the best timing accuracy^14^. Minimising the asynchrony variability has been suggested to facilitate close temporal coordination in joint actions, by increasing one’s own predictability^25^. When multiple cues are available that define the same underlying beat, humans combine the sensory information to estimate the beat structure^23^,^26^,^27^. Importantly, the combination process is statistically optimal, described mathematically by a Bayesian framework. This is a phenomenon observed across a range of perceptual tasks ^e.g.^ 28,29 and results in the best estimate of the event (or object) properties. In terms of movement timing, this has been shown to reduce variability of asynchronies relative to having the same information about the cue from a single source^23^,^27^,^30^.

Making larger corrections to minimise asynchrony variance is often at the cost of increasing the variability of one’s movements in terms of the timing intervals between the repetitive actions (denoted here as the Inter-Movement Intervals, IMI)^14^. Conversely, minimising the variability of the IMIs often results in higher asynchrony variance. This suggests the possibility of two timing strategies: the first where we optimise the timing of our movements in relation to an external cue (e.g. an auditory rhythm or the movements of another person) or second, where we optimise our own (internal) movement variability (i.e. minimising IMI variability). It is currently unknown when an internal versus external optimisation strategy might be applied.

In a variety of group performances, we observe an external optimisation strategy. For instance, successful quartet performance requires each member to regulate the timing of his or her own tone onsets to coincide with those performed by the other members^31^. Similarly, cohesive timing is essential for victorious rowing eights. Here, each rower’s inter-stroke-interval is closely linked with those performed by rowers in the same and opposite side of the boat^10^. These examples illustrate the need for individuals to control their own movement timing relative to those of the other members in a group. In the quartet study, two contrasting strategies were observed. In one quartet, the asynchrony correction gains were approximately equal between all players. In the other quartet, the first violin acted more like a leader with reduced correction gains relative to the other players. Thus, the two quartets evidenced contrasting styles of correction. However, in this study all players were able to see and hear all other players. It is interesting to ask what would happen if performers’ timing cues were limited to just one or two other performers^31^.

Here, we have developed a novel paradigm to investigate group timing strategies when timing cues were limited to one or two other players. We configured a group of six individuals into two ‘chains’ (Fig. 1A), whereby timing information, originating from a Leader, was passed along the chains (i.e. sequential cues passed via two Followers in each chain) to an individual sat between the ends of both chains (the Integrator, who synchronised with parallel cues). We hypothesised an increase in movement timing variability along the chain, as each participant added his or her own variability to the preceding cue. In contrast, we expected that participants in the Integrator position would combine the cue information from both chains and hence demonstrate improved synchronisation performance based on the optimal cue integration theory^23^,^27^.

We found that within each chain participants appeared to focus on reducing their asynchrony variance. However, this was achieved by increasing their correction gain as the cue became more variable and this substantially increased the variability of their own movements. When participants were in the Integrator position, they switched to a different strategy, optimising the timing of their own movements, at a cost of increased asynchrony variability. We conclude that while cue integration will usually result in reduced asynchrony variability, if the conflict between the cue onsets becomes too great, i.e. the phases between the cues becomes so large as to make integration impractical, individuals switch strategy to minimise their own movement timing variance.

## Results

### Cue source variability reflects timing strategy

**Figure 1:**
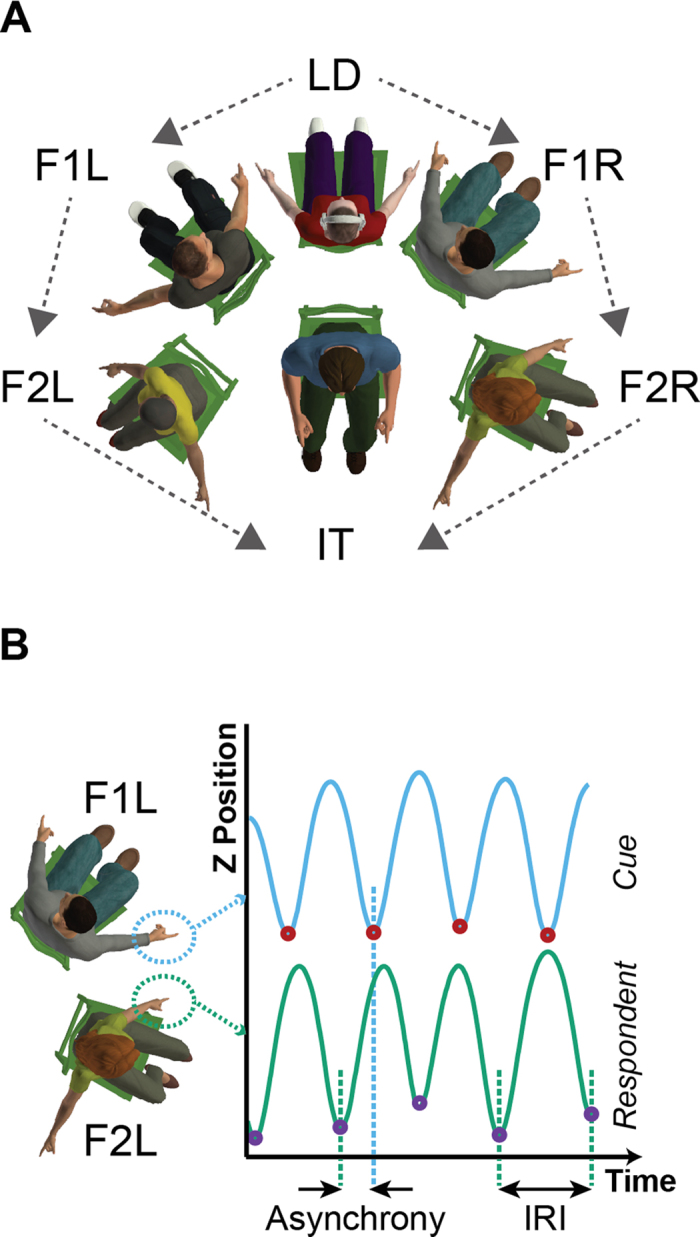
(**A**) Layout of positions. Six participants were seated in one of six positions, assigned randomly and rotated across trials. Participants seated in the lead position (LD) were instructed to synchronise up and down arm movements with an auditory metronome, presented over headphones. Participants who were sat in positions F1L and F1R were defined as Followers and instructed to synchronise their movements with the left and right arm movements of LD, respectively. Similarly, F2L synchronised their movements to F1L and F2R with F1R. Finally, participants in the Integrator position, IT, were set slightly back so they were able to observe and synchronise their own arm movements with those of both F2L and F2R simultaneously. Dashed arrows indicate the direction of information flow from LD round to IT (figure by Mark T. Elliott). (**B**) Data Analysis. A video motion capture system was used to track markers on the fingertips of each participant. Movement trajectories were subsequently measured in the vertical (Z) axis. Peak detection algorithms were applied to the trajectories to extract the times of the minimum positions of the movements (‘event times’, shown by circles). Pairwise comparisons were applied between each position and the adjacent position (or the metronome for position LD). In the example, we compare the event times from the left arm of position F2L (*respondent*, green trajectory) with that from the right arm of position F1L, who is providing the timing information (*cue*, light blue trajectory). Asynchrony is calculated as the difference between the event time of the respondent and the closest event time from the cue. A negative asynchrony indicates the respondent’s event time occurred earlier than the cue. The inter-response interval (IMI) is calculated as the duration between the respondent’s consecutive event times.

A group of six participants were seated in a quasi-circle formation facing outwards (Fig. 1A; see Methods for full details). The position a participant was seated in determined the role they took. The Leader (position LD, Fig. 1A) formed the head of the group and synchronised both left and right oscillatory vertical arm movements to an auditory metronome (inter-onset interval, IOI: 500 ms [Fast] and 800 ms [Slow], switched within a trial in either a slow-fast or fast-slow order). The four Followers (positions F1L, F2L, F1R, F2R; Fig. 1A) formed two chains either side of LD (two left, two right). The Followers synchronised their bimanual up and down movements of their lower arms with the adjacent person, i.e. left chain, the person to the right; right chain, the person to the left. Finally, the Integrator (position IT; Fig. 1A) was positioned between both chains, so they were able to observe both the left arm movements of F2L and right arm movements of F2R. The Integrator was instructed to time their arm movements so that they were in synchrony with both the left and right Follower movements. Each participant completed six trials in each position.

**Figure 2:**
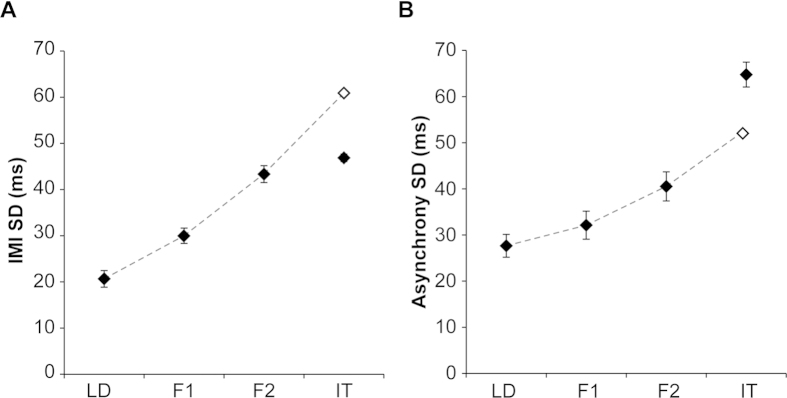
(**A**) IMI Variability. Variability in the movement intervals of participants increased as they were positioned further down the chain. However, in the IT position participants no longer showed a significant increase in IMI SD. Dashed line shows a polynomial fit (order 2) based on the points LD, F1 and F2 only. The open-diamond marker shows the predicted IMI SD for the IT position if the same timing strategies were applied as in positions LD-F2. Filled-diamonds show the actual data points – note the IT data marker is much lower than predicted. Error bars show standard error of the mean (SEM). (**B**) Asynchrony Variability. The variability of the asynchronies between each position and the adjacent position were calculated. Asynchronies increased further down the chain, albeit more gradually than that observed for IMI variability. However, in the IT position participants showed a substantial increase in their Asynchrony SD. Dashed line shows a polynomial fit (order 2) based on the points LD, F1 and F2 only. The open-diamond marker shows the predicted Asynchrony SD for the IT position if the same timing strategies were applied as in positions LD-F2. Filled-diamonds show the actual data points – note the IT data marker is much higher than predicted. Error bars show SEM.

We predicted that participants would show increasing asynchrony and IMI variability, the further along the chain they were positioned, due to participants adding their own variability to the timing information as it progressed along the chain. We did indeed see an effect of Position on both IMI standard deviation (F(3,15) = 68.55, p < 0.001) and asynchrony standard deviation (F(3,15) = 106.43, p < 0.001). However, post-hoc analyses revealed differences between the two measures (Fig. 2). Significant increases in IMI variability (Fig. 2A) were observed between the LD position and the Follower positions (F1, p = 0.015; F2, p < 0.001) and also between Follower positions (F1 to F2, p = 0.003). Importantly, we observed no significant increase in IMI variability between IT and the F2 positions (p > 0.999; Fig. 2A). This suggests that in the IT position, participants applied a different timing strategy that suppressed the increase in IMI variability observed across the other positions. This change in strategy was emphasised through further analysis of the asynchronies. Participants showed small increases in asynchrony standard deviation as they were positioned further along the chain (Fig. 2B), with a significant increase observed in position F2 (relative to F1, p = 0.006; LD, p = 0.038) but no significant change in variability between positions LD and F1 (p = 0.464). In contrast, asynchrony variability in position IT was substantially higher than the other positions (F2, p = 0.003; F1, p < 0.001; LD, p < 0.001). Overall, these results suggest that when participants were timing movements to a single cue input (i.e., in positions LD, F1, F2) they used a strategy that focussed on keeping asynchrony variance low. However, when positioned in the IT position, participants had to time movements to two sources of information that were potentially conflicting in phase. To deal with the conflicting cues, it appears that participants switched to minimising their own internal variability – that is reducing the variability of their IMIs at the cost of substantially increasing asynchrony variability to the cues. We observed the same strategy across positions for both fast and slow tempos, albeit with an overall increase in both IMI and asynchrony variability at the slow tempo (IMI: F(1,5) = 84.36, p < 0.001; Asynchrony: F(1,5) = 21.77, p = 0.006) as expected^32^,^33^.

We additionally investigated if the side at which participants were positioned in the chain (left-chain, right-chain) affected their timing performance. There were no significant effects of Side on either asynchrony or IMI variability, showing both left and right sides were moving with similar timing statistics. However, there was a Position x Side interaction for IMI standard deviation (F(3,15) = 60.47, p = 0.013) resulting from an increased variability observed at position F2R compared to F2L. We suggest this emergence of a higher variability in the F2R position is likely due to the analysis of participants’ non-dominant hand on this side (all but one were right handed).

### Reproduction of cue intervals

In addition to analysing the variability of the timing responses, we compared the mean asynchrony and IMI across positions for both fast and slow tempos and left and right chains. We found that as expected, participants were able to match the tempo of their movements with the metronome source regardless of their position in the chain (i.e. no significant effects of mean IMI for position or side). Hence, while IMI variability increased, the overall mean IMI remained true to the source.

**Figure 3:**
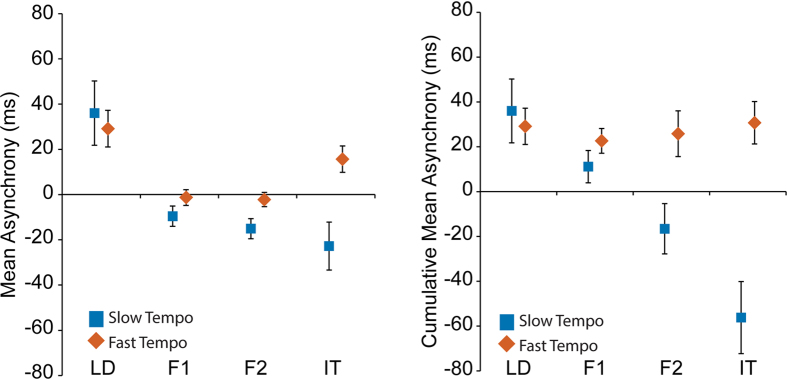
(**A**) Mean Asynchrony. The mean asynchrony for each position was calculated relative to the movements produced by the adjacent position towards LD. LD’s asynchronies were calculated relative to an auditory metronome. Error bars show SEM. (**B**) Cumulative Mean Asynchrony. The mean asynchronies for each position were all calculated relative to the original metronome beat onsets presented to LD. This gives the cumulative asynchrony along the chain. Error bars show SEM.

We found that mean asynchronies were positive in the LD position relative to the auditory metronome beats they heard (Fig. 3A), meaning movement event times were later than the beat onsets. The majority of sensorimotor synchronisation experiments report negative asynchronies when timing movements to an auditory metronome. Therefore, it appears in the LD position, participants weren’t synchronising the minimum position of their arm movements with the metronome beat, but some point earlier in the trajectory, for example the point of peak velocity^34^. The remaining positions showed asynchronies closer to zero, but varied by position and tempo (interaction, F(3,15) = 7.15, p = 0.003; Fig. 3A). To get a clearer picture of how the mean asynchrony developed along the chain we calculated the cumulative mean asynchrony. That is participants’ mean asynchrony at each position relative to the source metronome cue (rather than relative to the adjacent individual’s event times). We observed a clear interaction between the metronome tempo and the position (F(3,15) = 15.69, p < 0.001; Fig. 3B). For the Fast tempo, the positive asynchrony that emerges from the LD position remains almost the same regardless of position, with no difference between LD and IT (paired t(5) = -.11, p = 0.917). In contrast, the slow tempo metronome appears to invoke a negative asynchrony in all positions following LD such that cumulative asynchronies become more negative further down the chain (Fig. 3B; LD to IT, paired t(5) = 3.65, p = 0.015).

### Application of the Linear Phase Correction Model

To further understand the mechanics of the changes in the timing statistics according to the position in the chain, we applied a linear phase correction model to the experimental data^35^,^36^ (see Supplementary materials S1 for a full model description, online). This model has been shown to accurately describe the temporal correction process observed in sensorimotor synchronisation^14^. In particular, the model assumes the observed variance in the IMIs and asynchronies originates from two independent noise sources, namely the timekeeper and motor noise. The timekeeper noise represents the variability in the internal representation of the cue intervals observed, whereas the motor noise represents the variability resulting from executing the movements. Based on these two sources of variability, it is inevitable that there will be an error between the time of one’s own movement and the onset time of the cue. Therefore, the model describes a correction process that results in a temporal adjustment of the current movement that accounts for a proportion of the timing error made on the previous movement (i.e. the last perceived asynchrony). The proportional scaling parameter is termed the correction gain, denoted as *α* and is typically a value between zero (no correction made) and one (full correction made). Here, we implemented the bounded generalised least squares (bGLS)^37^ method to estimate the three model parameters, timekeeper variance 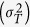, motor variance 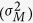 and correction gain (*α*).

**Figure 4:**
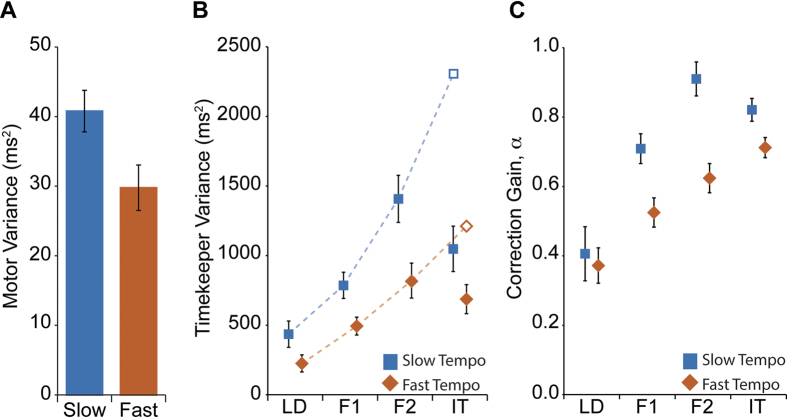
Parameters from a linear phase correction model were estimated from each participant’s asynchrony and IMI time series data. (**A**) Motor Variance. Estimates of motor variance remained relatively constant across positions. However, movements in the slow tempo conditions showed higher motor variance compared to estimates from the fast tempo conditions. Error bars indicate SEM. (**B**) Timekeeper Variance. As with motor variance, timekeeper variance was higher at slow movement tempos (blue square markers) than for fast movement tempos (orange diamonds). Estimates of timekeeper variance also showed increasing values further along the chain, indicating sensitivity to higher cue variance generated by the adjacent position. Moreover, as observed for IMI variability, we saw no significant increase in timekeeper variance in position IT, with actual estimates (filled symbols) being much lower than the predicted value (open symbol marker). Dashed lines show polynomial fits (order 2) based on the points LD, F1 and F2 only. Error bars are SEM. (**C**) Correction Gain. Estimates of correction gain indicate that participants actually increased their corrections as they moved further down the chain. Additionally, correction gains were larger in the slow tempo (blue squares) than the fast tempo (orange diamonds) conditions. Once again, we observed evidence in a change of strategy in the IT position compared to the other positions in the chain. Error bars are SEM.

We found that while motor variance was higher in the slow tempo condition than the fast tempo (F(1,5) = 10.49, p = 0.023; Fig. 4A), there was no significant change amongst positions. Therefore, the increase in timing variability that we observed as participants were positioned further along the chain was captured by the timekeeper variance estimates (F(3,15) = 14.39, p < 0.001; Fig. 4B). Moreover, we saw a drop-off in the timekeeper variance at the IT position, with no significant difference observed between IT and F2 (p = 0.184; Fig. 4B). As with the motor variance, the slower tempo increased timekeeper variance by a similar amount across positions (F(3,15) = 67.01, p < 0.001; Fig. 4B). These results suggest that the increased variability observed in the external cue is impacting on an individual’s internal timekeeper variability. However, by having the availability of two information sources, when participants are positioned in the IT position they appear to be combining the cue information^26^,^27^,^28^,^29^, hence reducing their own internal timekeeper variability.

The increasing cue variability along the chain also impacted on participants’ timing corrections with the correction gain increasing along the chain (F(3,15) = 13.39, p < 0.001; Fig. 4C). It appears therefore, that to remain in synchrony participants had to increase their correction as the variability of the cue increased. As higher correction gains increase IMI variability^14^, this result complements the suggestion that participants chose a strategy of maintaining a low asynchrony variability at the cost of increased IMI variability. In addition to measuring correction gains between adjacent positions, we extended the analysis to all pair-wise position combinations. We found the highest gain for each position was always associated with the adjacent position (see Supplementary Material, Fig. S4.1 online), confirming the flow of timing information along the chains. Non-adjacent positions showed consistently lower gains (not zero due to the correlation between movements within the group).

According to sensory integration theory, integrating the two cue sources in a statistically optimal fashion will result in a best estimate of the underlying timing information and hence minimise the variance in the cue^28^. While participants showed an increase in IMI variability as they were positioned further down the chain, in the IT position they showed no change in IMI variability. This evidence from our results suggests that in the IT position participants were integrating the timing intervals provided by F2L and F2R and hence minimising the variance in their own timing intervals. To test this further we used a nested linear model to examine if the timing intervals of IT could be significantly better predicted, using the time interval history of both F2L and F2R together compared to the time interval history of just one side (i.e. F2L *or* F2R; see Materials and Methods). Participants’ IMIs in the IT position were regressed against corresponding participant IMIs in the F2L position (Reduced model) and then F2L and F2R IMIs combined (Full model). The same was done for F2L and F2R intervals versus just F2R intervals. Comparing the sum squared errors of the residuals between the Full and Reduced models we calculated an F-statistic and p-value. Using a significance level threshold of p = .01, we found that the Full model was significantly better at predicting the IT intervals than the Reduced model for all participants (see Table 1) in both Fast and Slow conditions.

## Discussion

We have introduced a novel group timing paradigm in which a group of six participants were seated in a quasi-circle formation facing outwards (Fig. 1A). Participants’ roles were defined by position on the circle relative to one person designated as the leader (LD). Two chains comprising two followers on left and right side of the leader (F1L, F2L; F1R,F2R) were restricted to viewing timing cues from one other member further up the chain. At the end of the two chains, an integrator (IT) viewed timing cues from the second and last member of the two chains. The aim was to investigate how presentation and reliability of a sensory cue influences movement timing strategies. We applied a linear phase correction model^14^,^15^,^35^,^36^ to estimate how participants’ timekeeper and motor variances were affected by their position and role in the chain. Our results showed that the timing variability of a source cue is captured by participants’ synchronisation performance and as such, their timekeeper variance increased the further along the chain they were positioned. In contrast, their motor variance stayed relatively constant regardless of position.

Turning to correction gain, it has been shown previously that increased source cue variability reduces error correction gain in finger tapping paradigms^23^. However, the present work found the opposite, an increase in error correction gain with increased cue variability. Importantly, our results showed that participants switched timing strategies depending on the task demands. Specifically, those in the IT position were instructed to synchronise movements based on the two timing cues provided by participants in the F2L and F2R positions. We found that in the IT position participants reduced their own movement variability (in terms of the measured IMI variance), at the cost of increased asynchrony variability to each of the external cues. This is in contrast to a strategy that appeared to focus on maintaining a low asynchrony variability when sat in the other positions. We propose that participants reverted to this self-optimisation strategy when the integration of both source cues may have been unattainable.

In our study, the lead position (LD) synchronised their movements to an auditory metronome, while the remaining positions synchronised to continuous visual cues generated by the arm movements of the adjacent person. Synchronising movements to an auditory metronome is a simple task and results in low asynchrony variability between the movement events and the metronome. In contrast, participants often report difficulty synchronising with a discrete visual metronome (flashing light) during finger tapping tasks^38^. However, in real life scenarios we are more likely to move in time with a continuous visual stimulus with spatial as well as temporal information, for example coordinating movements with a dance partner or a fitness instructor. Temporal variability of movements is shown to be much reduced compared to synchronising with discrete visual cues^39^, albeit still not quite reaching the performance of an auditory metronome^40^.Consequently, as one would predict, participants showed lowest variability in the LD position for both IMI and asynchrony variability measures. However, as well as a change of modality, participants in subsequent positions were synchronising to cues with reduced regularity, due to the adjacent person’s own timing variability affecting the cue. As we initially predicted, we observed an accumulative effect with the irregularity of the cue increasing the respondents IMI variability relative to the preceding position. Using a linear phase correction model^35^,^36^ fitted to the empirical data, we were surprised to observe an increase in timekeeper variance as participants were positioned further along the chain. It therefore appears that an individual’s own internal timekeeper reliability is directly affected by the external cue reliability. One possible explanation for this observation is that high variability in the cue resulted in participants performing period correction^13^ as well as phase correction. As our model only considered phase correction, then this extra correction process could have inflated the estimates of the variance in the timekeeper. The estimates of motor noise on the other hand, remained constant along positions.

One way for participants to reduce the rate of increase in IMI variability along the chain would be to reduce their correction gain. That is, for higher cue irregularities the correction is reduced as to maintain stable timing in one’s own movements, at a cost of reducing synchrony with the cue. This reduction in correction gain has been observed in perturbation studies where a single metronome interval is lengthened or shortened unexpectedly^34^. Metronomes presented visually (hence a less reliable cue) resulted in much reduced corrections to the perturbation than when the same perturbation was applied to an auditory metronome^41^. We expected that participants might implement this strategy in our paradigm such that we would observe lower correction gains progressing along the chain as the cue became more irregular. We in fact observed the opposite: participants increased their correction gain further along the chain. We suggest this difference originates from confidence in the cue itself. In our study, while the variability in the visual cues is high, it remains consistent throughout the trial and therefore participants have a reasonable level of confidence in the temporal properties and hence apply a high correction to overcome the irregularity. In contrast, a random perturbation is unexpected and the central nervous system (CNS) must consider whether this is a true environmental change or just an estimation error within the sensory system^42^. The high variability of a visual cue provides less confidence in the sudden perturbation and hence the CNS lowers the correction gain to avoid over-compensation^42^. It therefore appears that participants were following task instructions and synchronising their arm movements with those of their adjacent partner, increasing correction gain in order to minimise asynchrony variability. The knock on effect of minimising asynchrony variability was an increase in the variability of the IMIs. The question remains as to whether this strategy could remain in longer chains: by aiming to maintain synchrony, larger correction gains would potentially be required to compensate ever-increasing IMI variability. Eventually, it would be expected that this would result in instability in the system as gains equal or exceed full correction^14^,^15^ and can no longer minimise asynchrony due to increasingly large irregularity in the timing cues.

The results we have described so far have excluded the IT position, which we found applied a different timing strategy to manage the demands of synchronising with two variable cues simultaneously. In contrast to the observed increase in IMI variability across the chain of Followers, no increase was found for IT, relative to the adjacent left and right F2 positions. However, asynchrony variability was found to be substantially higher in IT than in the other positions. Research investigating synchronisation to multiple cues within and across sensory modalities found evidence of cue integration through reduced variability in the asynchronies^23^,^26^,^27^,^30^. With the two cues relatively closely matched in phase, the reduced asynchrony variance observed could be predicted and shown to be statistically optimal using a model of Maximum Likelihood Estimation^27^. The authors reported a break down in optimal integration when they presented participants with a highly irregular metronome beat. In a further study, the same authors showed that the CNS determines whether to integrate two sources of rhythmic cue information (or alternatively treat them independently) based on the phase separation and regularity of the individual sources^26^. Both these studies, report on the change in asynchrony variance, but not IMI variance. We suggest that in our experiment, participants positioned in F2 within the left and right chains were producing movements that were substantially out of phase with each other. Therefore, participants seated in IT were unable to integrate the cues in order to reduce their asynchrony variability, similar to what was observed in previous studies^26^,^27^. In contrast to these earlier studies we also measured IMI variance and were able to observe a change in timing strategy due to the conflicting cues. That is, it appeared that participants seated in the IT position, optimised the variability of their own movements, such that we saw no increase in IMI variability compared to the F2 positions. Moreover, there was no further increase in timekeeper variance in position IT compared to F2. Importantly, this suggests that participants in position IT were still using both cues from left and right F2 positions, but potentially integrating the interval (rather than phase) information to reduce their timekeeper variability and subsequently the variability of their own movements. We provided further evidence of this using a nested linear model. For all participants, we found that their IMIs in the IT position were significantly better predicted using a full model (consisting of the associated left and right F2 IMIs) than a reduced model using the F2 IMIs from just one side.

In summary, the present work demonstrates a novel methodology to study synchronous group performance. Our results provide evidence for the employment of two different timekeeping strategies in group synchronisation: external versus internal optimisation. We have shown that timing variability of a cue source is reflected in the participants’ synchronisation performance, as the timekeeper variance increased with position along the chain. We further found, that participants switched to a self-optimisation (internal) timekeeping strategy when synchronisation to both source cues simultaneously may have been impractical. Here, participants reduced their own inter-movement-interval variability to keep in time with both source cues’ timing intervals. We conclude that people are able to flexibly switch timekeeping strategies to maintain task demands and thereby continue to optimise their movement timing performance even in challenging group scenarios. Current research in our lab is directed towards exploring the use of different modalities (e.g. audio, haptic) in groups of varying size to examine the generalisability of the findings reported here.

## Materials and Methods

### Participants and Design

Six participants (3 female, one left-handed, aged 30–41 with a mean age 37.2) were recruited from staff and students at the University of Birmingham and they provided written informed consent. All participants were healthy and had normal or corrected-to-normal vision. Only one participants had previous musical experience (playing the guitar) and all participants knew each other on a professional level. The experimental protocols were approved by the University of Birmingham Ethical Review Committee and complied with the Declaration of Helsinki. The experiment had a 2 (Tempo: Fast (500 ms metronome interval), Slow (800 ms metronome interval)) x 2 (Side: Left chain, Right chain) x 4 (Position: LD, F1, F2, IT) repeated measures design.

### Experiment Setup and Apparatus

Participants were seated on chairs arranged in a quasi-circle with participants facing outwards (Fig. 1A). The gap between chairs was 20 cm. In total, there were three roles; Leader (LD), Follower (of which there were four) and Integrator (IT). The roles for the chain on the left hand and right hand side of LD were Follower 1 (F1L – left chain; F1R- right chain) and Follower 2 (F2L – left chain; F2R – right chain). Closing the circle formation, position IT was located slightly behind F2L and F2R to allow him or her to see the movements from positions F2L and F2R simultaneously. Metronome presentation for LD was controlled using the MatTAP toolbox^43^ in Matlab^44^, operating through a data acquisition device^45^ and presented via noise cancelling headphones. The volume of the headphones was set to a level where other participants reported not to hear any sound. Those in the Follower and IT positions wore soft silicone earplugs to suppress any background noise that may be produced by participants’ movements.

Movement kinematics were recorded at 200 Hz using a twelve camera motion tracking system^46^. Two 20 mm diameter spherical reflective markers were attached to each participant’s fingertips (left and right index finger). Movement trajectories in the vertical (z-axis) direction were used for analysis.

### Task and Procedure

The task was explained to the six participants along with instructions on how movements were to be synchronised in each of the positions. Two practice trials were performed before the experimental trials began. All participants were instructed to synchronise the lowest point of their downward lower arm movement with the associated cue for that position. In position LD, participants synchronised movements to an auditory metronome presented over headphones. LD was instructed to close their eyes at all times to avoid additional visual timing cues potentially resulting from their own and other group members’ movements. In the Follower positions, participants were instructed to synchronise their bi-manual oscillatory arm movements with their adjacent partner’s arm closest to them (see Table 2). Participants seated in position of IT were instructed to synchronise their bi-manual arm movements with both F2L’s left arm and F2R’s right arm simultaneously. Throughout the synchronisation task all participants were told to move their left and right lower arm synchronously and to point with their index fingers.

The experiment consisted of six blocks with six trials each, in total 36 trials. At the start of every new block the experimenter assigned the role to which participants were allocated to by randomised permutation (see Supplementary Materials S3), so that each member was in every position once. In each trial, participants in position LD were presented with thirty ‘fast’ inter-onset-interval (IOI) beats and thirty ‘slow’ IOI beats. The metronome IOIs varied across trials to minimise participants learning the beat. For the fast tempo, the IOI was a randomly selected value between 450 ms and 550 ms, while for the slow tempo, a random value between 720 and 880 ms was selected. The beat remained isochronous within slow and fast segments of the trial, while the tempo switch was a step change at beat 31. The order of the fast/slow tempo presentation was counterbalanced and randomized within each block, with three trials starting with fast IOIs followed by slow IOIs, and three trials in slow-fast presentation. For analysis purposes, we only considered the movement onsets during the steady state of a trial, and therefore, discarded the two movement onsets preceding the tempo change and the subsequent three following movements, to allow for tempo adjustments. Validating this approach, we found no difference between Fast-Slow and Slow-Fast presentations during preliminary analyses, and therefore simplify the analysis by only comparing Slow and Fast tempo segments without the additional variable of presentation order. Participants were not explicitly informed when the tempo change would occur, to ensure participants’ undivided attention to the arm movements of their lead throughout trails. However, after familiarising themselves with the task during the initial practice trials, we expected that participants were conscious of the fact that a tempo change would take place within each trial. On completion of the task, participants were debriefed as to the purpose of the experiment.

### Analysis

For each trial, we recorded the vertical trajectory of each participant’s fingertip movements (200 samples per second) and subsequently low pass filtered the data (cut-off frequency: 20 Hz) using a zero-phase Butterworth filter. We used a peak detection algorithm in Matlab to extract the event times at which the movements were at their lowest vertical positions during each oscillatory cycle. We further recorded the onset times of the auditory metronome beats, which were sampled in synchrony with the motion capture video frames. IMIs were calculated as the durations between the event times of the movements (Fig. 1B). Asynchronies were calculated by subtracting the relevant *cue* event time from the *respondent’s* corresponding event time (see Table 2; Fig. 1B). For example (as shown in Fig. 1B), in the case of position F2R we subtracted position F1R’s right hand event times (the *cue* event times) from position F2R’s left hand event times (the *respondent* event times). In addition, we calculated the respondent asynchronies for each position relative to the source metronome beats to get a measure of the cumulative asynchrony along the chain (as presented in Fig. 3B). To account for slight movement tempo differences between participants each respondent’s onsets were first aligned to the cue onsets. That is, for every cue event time we matched the closest respondent event time to that beat. This resulted in a one-to-one comparison between the cue and respondent event times (cf.^43^).

### Statistical Analyses

We analysed the results using a repeated measures design with the factors: Tempo (Fast, Slow), Side (Left chain, Right chain) and Position (LD, F1, F2, IT). We analysed the mean and standard deviation of IMIs, asynchronies and cumulative asynchronies for each condition. All analyses met the assumption of sphericity. The p-values of post-hoc analyses were Bonferroni corrected. Results were assumed significant where p < .05, unless otherwise stated.

### Computational Analyses: Estimation of linear phase correction parameters

A linear phase correction model accurately describes the temporal corrections observed when individuals are timing movements in synchrony with an external cue^14^. The model can be used to estimate the correction gain, i.e. the proportion of the preceding asynchrony which the current movement has corrected. In addition, the independent variance components originating from the ‘timekeeper’ (i.e. one’s internal representation of the beat) and the production of the motor action can be estimated. Here we used the bounded Generalised Least Squares (bGLS) method^35^,^36^ to fit the linear phase correction model to the empirical data. In the supplementary materials S1 we describe an extension to the bGLS method we developed to firstly, account for the bimanual (left and right) arm movements’ participants performed and secondly, to accommodate the special case of the IT position, where two timing sources were available.

The event times of the respondent’s left and right arm movements along with the related cue event times were used to estimate the respondent’s correction gain (*α*), timekeeper variance (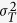) and motor variance (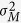) for each position and tempo. Values estimated for left and right Follower positions were averaged together due to no observed differences in the left and right chains from the experimental analyses. Statistical tests were therefore based on a repeated measures design with the factors of Tempo (Fast, Slow) and Position (LD, F1, F2, IT). Given the relatively new approach of the bGLS method, we compared the motor variance estimates with those estimated from an alternative method calculated using the variances of left and right arm timing intervals^47^.

### Computational Analyses: Hierarchical modelling to test for integration in position IT

According to optimal cue integration, based on a maximum likelihood estimation (MLE) approach, the resulting variance in the observed variable will always be lower when information from two cues are combined than either of them alone. The experimental results suggested participants in the IT position were integrating the timing information from both left and right chains. However, evidence of integration came from the reduced variability of the timing intervals, rather than reduced asynchrony variability observed in previous studies^30^,^48^.

We tested for evidence that participants were using cue information from both chains, rather than just one chain, using a nested model approach. For each participant, the IMI data from each trial in the IT position was concatenated into a single time series. The corresponding IMI data from participants in positions F2L and F2R were similarly concatenated into single time series. Participants’ IMIs in the IT position were then regressed against corresponding participant IMIs in the F2L position (Reduced model) and then F2L and F2R IMIs combined (Full model). Thus, the reduced model is defined as:


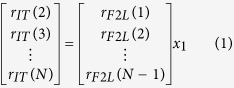


while the full model is defined as:


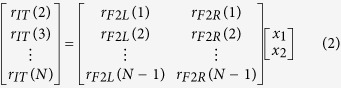


where *r*_*IT*_, *r*_*F2L*_, *r*_*F2R*_ are the IMIs from the IT, F2L and F2R positions respectively, *x*_*1*_ and *x*_*2*_ are the regression coefficients and N is the total number of IMI responses.

The reduced model in (1) is hence, embedded in (2). It is certain that the full model will reduce the unexplained variance in relation to the reduced model. However, we needed to test if the reduction in unexplained variance was significant, hence suggesting that the IT IMIs are dependent on both left and right chains. To do this we use an F-test to compare the sum-squared error for the reduced model (*SSE*_*Reduced*_) with the full model (*SSE*_*Full*_)^49^:


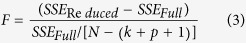


where *k* is the number of regression coefficients in the reduced model (one in this case) and *p* is the number of additional coefficients in the full model (one). Using the *F* statistic, we were able to look up the corresponding *p*-value. We judged the full model to be significantly better at predicting the IT IMIs when *p* < .01.

## Additional Information

**How to cite this article**: Honisch, J. J. *et al.* Cue properties change timing strategies in group movement synchronisation. *Sci. Rep.* **6**, 19439; doi: 10.1038/srep19439 (2016).

## Supplementary Material

Supplementary Information

## Figures and Tables

**Table 1:** A linear nested model was used to test whether the Integrator (IT) combined both of his/her target cues.

Participant	Fast				Slow			
	F2L vs F2L + F2R		F2R vs F2R + F2L		F2L vs F2L + F2R		F2R vs F2R + F2L	
	*F-val*	*p *<* 0.01*	*Fval*	*p *<* 0.01*	*F-val*	*p *<* 0.01*	*Fval*	*p *<* 0.01*
1	87.34	Yes	243.97	Yes	58.00	Yes	111.53	Yes
2	50.64	Yes	288.47	Yes	29.69	Yes	155.28	Yes
3	50.41	Yes	24.72	Yes	76.69	Yes	28.21	Yes
4	147.37	Yes	9.30	Yes	58.19	Yes	67.78	Yes
5	246.59	Yes	422.76	Yes	115.22	Yes	110.54	Yes
6	52.30	Yes	106.39	Yes	165.81	Yes	66.78	Yes

IT’s IMIs were regressed against the IMIs from just a single side (F2L *or* F2R; Reduced model) and then the IMIs of both F2R *and* F2L (Full model). The F-statistics and p-values from the difference in sum squared errors of the residuals for each participant in slow and fast interval durations are presented. The results show that the full model better predicts each the IT IMIs for every participant.

**Table 2:** Target cue. Each position within the left and right chain synchronised their oscillatory arm movements to a designated target cue provided by their adjacent partner.

*Position*	Left Chain		Right Chain	
	*Respondent Events*	*Cue Events*	*Respondent Events*	*Cue Events*
LD	Left Arm	Metronome	Right Arm	Metronome
F1	[F1L] Right Arm	LD Left Arm	[F1R] Left Arm	LD Right Arm
F2	[F2L] Right Arm	F1L Left Arm	[F2R] Left Arm	F1R Right Arm
IT	Right Arm	F2L Left Arm	Left Arm	F2R Right Arm

The table illustrates the target cue for each position. For data analysis, the asynchronies were calculated subtracting the relevant cue time event form the corresponding event time performed.
