# Supplementary material for: Cue properties change timing strategies in group movement synchronisation
Source: Sci Rep. 2016 Jan 19;6:19439. doi: 10.1038/srep19439 (PMC4726081; doi:10.1038/srep19439)
Supplement: Supplementary Information [file srep19439-s1.pdf]

Supplementary Material

Cue properties change timing strategies in group movement synchronisation

Juliane J. Honisch*[1,5], Mark T. Elliott[2,5], Nori Jacoby[3,4], Alan M. Wing[5]

1. School of Psychology and Clinical Language Sciences, University of Reading, Reading, RG6 6AL, UK.
2. Institute of Digital Healthcare, University of Warwick, Coventry, CV4 7AL, UK.
3. Department of Brain and Cognitive Sciences, Massachusetts Institute of Technology, Cambridge, Massachusetts, USA.
4. Music Department, Bar Ilan University, Ramar Gan, Israel.
5. School of Psychology, University of Birmingham, Edgbaston, B15 2TT, UK.

*Corresponding author. Email: J.J.Honisch@reading.ac.uk

Table of Contents

Supplementary Material 1

1. Supplementary Information S1 3

1.1 Estimating bimanual synchronization model with the bGLS method 3

1.2 Algorithm 1 5

1.3 Integrator model 7

2. Supplementary Information S2 8

2.1 Comparison of bGLS motor variance estimates with an alternative method 8

2.2 Calculating Motor Variance 8

3. Supplementary Information S3 10

3.1 Randomised permutation of positions 10

3.2 Test for practice effect across positions 10

4. Supplementary Information S4 11

4.1 Mean alpha correction gains relative to all positions 11

1. Supplementary Information S1

1.1 Estimating bimanual synchronization model with the bGLS method

The bGLS method provides a robust and efficient way to estimate the parameters of synchronization models[1,2]. The method is based on the standard generalized least squares approach[3], modified to the specific structure of synchronization models[2]. We develop here a bimanual version of this method.

We denote by O_t^R the subject response at time *t*. We denote by O_t^S the reference stimulus at time *t*, that is either the metronome onsets (lead position) or the onsets of the adjacent partner to which the participant is synchronizing their movements.

We denote by S_t,R_t and A_t as the inter-stimulus ,inter-movement interval and asynchrony at time t: $S_t = O_t^S - O_{t-1}^S$, $R_t = O_t^R - O_{t-1}^R$, $A_t = O_t^R - O_t^S$

The Vorberg and Wing model[4], analyzed in Vorberg and Schulze [5], can be written in this notation as:

(1) $R_{t+1} = (-\alpha)A_t + t_0 + T_t + M_t - M_{t-1}$

Where t_0 is the base tempo, T_t and M_t are the timekeeper and motor variances respectively, and var(T) = σ_T^2, var(M) = σ_M^2.

The model can be extended to bimanual movement, assuming that the timekeeper component is shared between hands, and that the motor variance of the two hands is uncorrelated.

This can be written with the following equations:

(2) $R_{t+1}^L = (-\alpha)A_t^L + t_0 + T_t + M_t^L - M_{t-1}^L$

(3) $R_{t+1}^R = (-\alpha)A_t^R + t_0 + T_t + M_t^R - M_{t-1}^R$

Where R_{t+1}^L and R_{t+1}^R are the next inter-response intervals of the left and right hand respectively; and A_t^L and A_t^R are the current asynchrony of the left and right hand (compared with the reference stimulus), respectively; M_t^L, M_t^R and T_t are the left motor noise, the right motor noise, and the shared (central) timekeeper noise, respectively.

Note that:

(4) $\mathrm{Var}(R_{t+1}^L + \alpha A_t^L) = \mathrm{var}(R_{t+1}^R + \alpha A_t^R) = \sigma_T^2 + 2\sigma_M^2$

(5) $\mathrm{Cov}(R_{t+1}^L + \alpha A_t^L, R_{t+1}^R + \alpha A_t^R) = \sigma_T^2$

The model's equation directly implies the following equation, obtained by averaging equations (2) and (3):

$$(6)\ \frac{R_{t+1}^{L}+R_{t+1}^{R}}{2} = -\alpha\frac{(A_t^L+A_t^R)}{2} + t_0\ + T_t + \frac{M_t^L+M_t^R}{2} - \frac{M_{t-1}^L+M_{t-1}^R}{2}$$

Note that this equation has exactly the same structure as equation 1, but with averaged asynchrony and motor noise values.

Without loss of generality we can assume that A_t^L, A_t^R, R_t^L and R_t^R have all zero mean. This can be always achieved by performing a pre-process where we reduce from each of the variables its empirical mean (for example: $A_t^L \rightarrow A_t^L - \frac{1}{N}\sum_{t=1}^{N} A_t^L$). We can therefore directly apply the bGLS method by rewriting equation (6) in matrix notation:

$$(7)\ y = Bx + Z$$

Where:

$$y = \begin{bmatrix} \frac{R_3^L + R_3^R}{2} \\ \vdots \\ \frac{R_{N+1}^L + R_{N+1}^R}{2} \end{bmatrix}, \qquad x = -\alpha,$$

$$B = \begin{bmatrix} \frac{A_2^L + A_2^R}{2} \\ \vdots \\ \frac{A_N^L + A_N^R}{2} \end{bmatrix}, \qquad Z = \begin{bmatrix} T_2 + \frac{M_2^L + M_2^R}{2} - \frac{M_1^L + M_1^R}{2} \\ \vdots \\ T_N + \frac{M_N^L + M_N^R}{2} - \frac{M_{N-1}^L + M_{N-1}^R}{2} \end{bmatrix}$$

The covariance matrix of Z is given by:

$$(8)\ \Sigma = \mathrm{COV}(Z) = \begin{bmatrix} \sigma_T^2 + \sigma_M^2 & \frac{-\sigma_M^2}{2} & & 0 & 0 \\ \frac{-\sigma_M^2}{2} & \ddots & & \ddots & 0 \\ & & & & \\ 0 & \ddots & & \ddots & \frac{-\sigma_M^2}{2} \\ 0 & 0 & & \frac{-\sigma_M^2}{2} & \sigma_T^2 + \sigma_M^2 \end{bmatrix}$$

Assuming we know σ_M^2 and σ_T^2, then the maximal likelihood estimator of $x = -\alpha$ is given by the GLS solution (Aitken 1935):

$$(9)\ x_{\text{estimated}} = (B^T\Sigma^{-1}B)^{-1}(B^T\Sigma^{-1}y)$$

If α is known than we can directly estimate σ_M^2 and σ_T^2 from equations (4) and (5).

Since in our case both alpha and the motor noises (σ_M^2 and σ_T^2) are unknown, we can use the following iterative bGLS algorithm (similar to Jacoby and colleagues [1]section 2.3.1). As explained in Jacoby and colleagues [1,2], it is important to further constrain the motor variance to the range $0 < \sigma_M^2 < \sigma_T^2$, otherwise the resulting estimate may be unreliable. Note that this assumption is empirically justified (see Wing[6]).

1.2 Algorithm 1

- Input: the vector y, and B
- *niter* a constant that determines the number of iterations.

Output: the estimated parameters$(\bar{\alpha}, \bar{\sigma}_T, \bar{\sigma}_M)$

- Start by setting $\Sigma_1 = I$ (the N by N identity matrix).
- Iterate the following equations *niter* times:

(i) Compute: $\alpha^n = -(B^T \Sigma_n^{-1} B)^{-1}(B^T \Sigma_n^{-1})y$

(ii) Compute: $d_t^{L,n} = R_{t+1}^L + \alpha A_t^L$, $d_t^{R,n} = R_{t+1}^R + \alpha A_t^R$.

(iii) Compute: $v^n = \frac{1}{2N}\sum_{t=1}^{N}\left[d_t^{R,n}d_t^{R,n} + d_t^{L,n}d_t^{L,n}\right]$

(iv) Compute: $w^n = \frac{1}{N}\sum_{t=1}^{N} d_t^{L,n}d_t^{R,n}$

(v) Estimate: $(\sigma_M^n)^2 = \frac{v^n - w^n}{2}$

(vi) Estimate: $(\sigma_T^n)^2 = v^n - 2(\sigma_M^n)^2$

(vii) Adjust $(\sigma_M^n)^2$ by decreasing it so that:
$0 < (\sigma_M^n)^2 < (\sigma_T^n)^2$.

(viii) Compute: $\Sigma_{n+1} = \left[(\sigma_T^n)^2 + (\sigma_M^n)^2\right]I + [\frac{-\sigma_M^n}{2}]\Delta$, When I is the N by N identity matrix and Δ is a N by N matrix with one on the two secondary diagonals and 0 elsewhere.

- The output is $(\bar{\alpha}, \bar{\sigma}_T, \bar{\sigma}_M) = (\alpha^n, \sigma_T^n, \sigma_M^n)$ computed at the last iteration *niter*.

Figure S1 shows simulation results that demonstrate that the method provides an unbiased estimate. We simulate 1000 iterations of the model for alpha=0.1,0.2,…,0.9,1 and for $\sigma_T^2 = 100, \sigma_M^2 = 25$. For each simulated dataset, we estimated the parameters using algorithm 1. The figure shows the mean estimates and standard errors (thick

line) compared with the true value (dashed line). The x-axis is the simulated alpha, and the y-axis is the estimated parameter. We can clearly see that the estimates are unbiased, and the estimation error is comparable with the uni-manual version (see Jacoby and colleagues [1,2]).

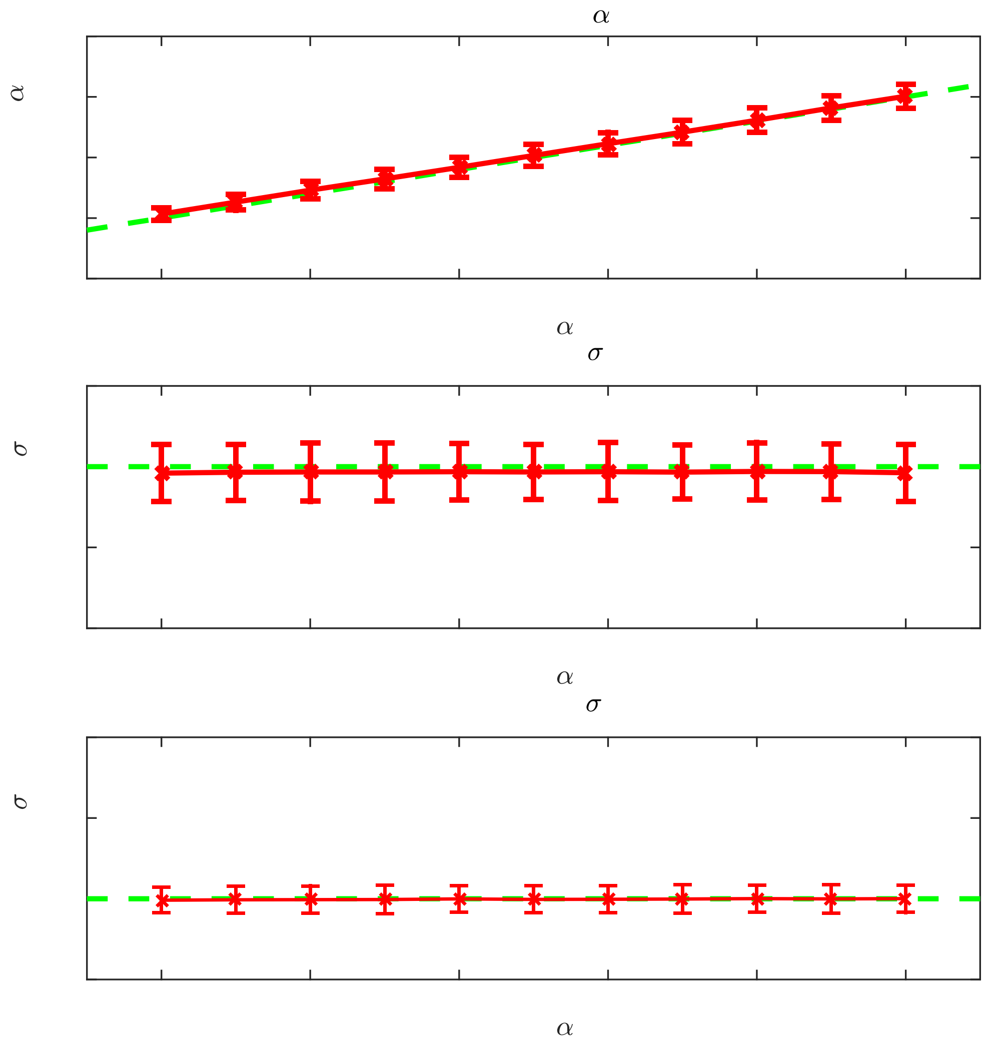

Figure S1.1

Simulation results of parameter estimation using algorthim 1. In these simulations, we scanned multiple values of alpha, while setting $\sigma_T^2 = 100$ *and* $\sigma_M^2 = 25$*. For each alpha we computed 1000 iterations of the simulation and estimated alpha (top figure, thick black line), the timekeeper variance (middle figure, thick black line), and the motor variance (bottom figure, thick black line). In each graph the ideal estimates were plotted as the dashed line.*

For example, in the top diagram the dashed line is defined by the equation "estimated α = true α". Error bars represent standard error of the estimates.

1.3 Integrator model

The integrator role in this experiment is an interesting case study of the recently developed research on ensemble synchronization (see review in Keller [7]). An ensemble version of the bGLS approach was also suggested in Jacoby and colleagues [1,2]. The extension of this model to the bimanual case analyzed above is relatively straightforward:

(10) $$R_{t+1}^{L} = (-\alpha_1)A_t^{1,L} + (-\alpha_2)A_t^{2,L} + t_0 + T_t + M_t^L - M_{t-1}^L$$

(11) $$R_{t+1}^{R} = (-\alpha_1)A_t^{1,R} + (-\alpha_2)A_t^{2,R} + t_0 + T_t + M_t^R - M_{t-1}^R$$

Where α_1 and α_2 are the phase correction constants that relate to each of the integrator reference stimuli, namely, to each of the subjects that the integrator should integrate; $A_t^{1,L}$ and $A_t^{2,L}$ are the asynchronies of the integrator left hand with respect to the two possible partners; $A_t^{1,R}$ and $A_t^{2,R}$ are the asynchronies of the right hand with respect to the two partners.

If we modify the previous algorithm by defining:

(12) $$B = \begin{bmatrix} \frac{A_2^{1,L}+A_2^{1,R}}{2} & \frac{A_2^{2,L}+A_2^{2,R}}{2} \\ \vdots & \vdots \\ \frac{A_N^{1,L}+A_N^{1,R}}{2} & \frac{A_N^{2,L}+A_N^{2,R}}{2} \end{bmatrix},\ x = \begin{bmatrix} -\alpha_1 \\ -\alpha_2 \end{bmatrix}$$

We can use the same algorithm to compute the integrator variables replacing step (ii) in the algorithm with the following definition, without further change in the algorithm.

(13) $$d_t^{L,n} = R_{t+1}^{L} + \alpha_1 A_t^{1,L} + \alpha_2 A_t^{2,L},\ d_t^{R,n} = R_{t+1}^{R} + \alpha_1 A_t^{1,R} + \alpha_2 A_t^{2,R}$$

While this method also provides reliable estimates, we did not include them here as they are almost identical to those with one subject and to the multiple person case study analyzed in Jacoby, et al.[1,2].

2. Supplementary Information S2

2.1 Comparison of bGLS motor variance estimates with an alternative method

The bounded general least squares (bGLS) approach was recently introduced by Jacoby and colleagues[1,2] to provide a highly flexible method for estimating the parameters of the linear phase correction model of sensorimotor synchronisation. We have taken the opportunity to use our data from this study to compare one of the parameters (motor variance) generated by the bGLS model to that estimated by an alternative method[7].

For the alternative motor variance estimate we exploited the availability of bimanual arm movement data and used the covariance between the inter-movement intervals (IMIs) of the left and right arms. Below, we give a brief overview of the methods and the results.

Calculating Motor Variance

Using the linear phase correction model[5,4], we can define the left arm intervals by:

(14) $$R_{t+1}^{L} = T_t + M_t^L - M_{t-1}^L$$

Where R_{t+1}^{L} is the observed left arm IMI, T_t is the common Timekeeper interval and M_t^L, M_{t-1}^L*1* are the left arm motor delays, respectively (see previous section).

Note that in this version we ignore the phase correction in the model of Supplementary Information S1.

Similarly:

(15) $$R_{t+1}^{L} = T_t + M_t^L - M_{t-1}^L$$

Where, $R_{t+1}^{L} =$ is the observed right arm IMI, and M_t^L, M_{t-1}^L are the left arm motor delays, respectively.

From this, we can infer that the asymptotic variance of the intervals can be defined as:

(16) $$\sigma_L^2 = \sigma_T^2 + 2\sigma_{M^L}^2 \text{ and,}$$

(17) $$\sigma_R^2 = \sigma_T^2 + 2\sigma_{M^R}^2$$

where σ_L^2, σ_R^2 are the measured variances of the left/right IMIs, σ_T^2 is the timekeeper variance and $\sigma_{M^L}^2$, $\sigma_{M^R}^2$ are the left/right motor variances, respectively.

From Vorberg & Hambuch's methods[7], we can estimate the timekeeper variance from the covariance between the left and right intervals. Therefore, we can then estimate the corresponding left/right motor variances as:

(18) $$\sigma^2_{M^L} = \frac{1}{2}(\sigma^2_L - cov(R^L, R^R))$$

Similarly:

(19) $$\sigma^2_{M^R} = \frac{1}{2}(\sigma^2_R - cov(R^L, R^R))$$

Results: Motor Variance

For motor variance, we found that while the bimanual estimation method gave higher estimates than the bGLS method for both fast and slow tempos, the difference was not significant (*F*(1,10)=4.55, p=.059; Fig. S2.1a). In addition, we found a significant correlation between all estimates (across all participants, conditions and positions) (*r* =.334, *p*=.020; Fig. S2.1b). These small differences result from the inclusion of the phase correction gain parameter in the estimates in the bGLS method.

(a).

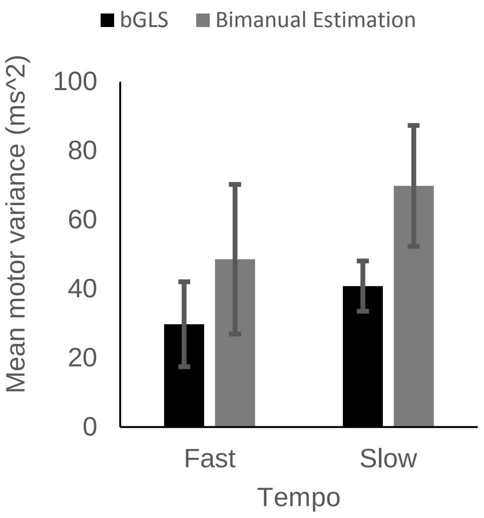

(b).

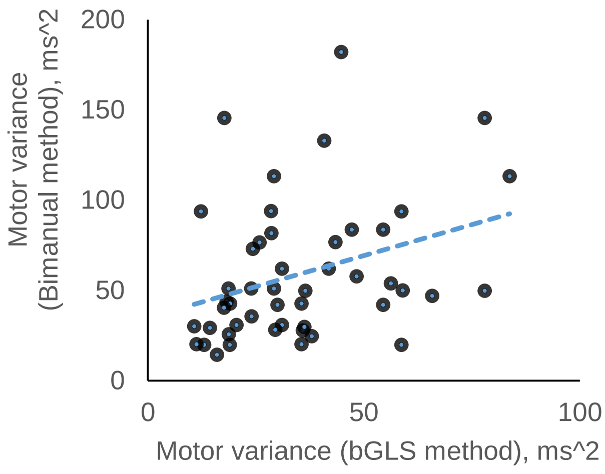

***Figure S2.1. (a)** Mean motor variance estimation for fast and slow tempo, using the bimanual estimation method versus bGLS. Error bars represent standard error of the mean. **(b).** Individual motor variance estimates plotted using bGLS versus bimanual estimation methods. Dashed line shows least squares line of best fit.*

3. Supplementary Information S3

3.1 Randomised permutation of positions

Participants were assigned to their positions by means of a randomised permutation, so that each participant was not synchronising to the same person at all times. However, given the limitations of the six positions available, some participants were synchronising to the same lead (cue) up to two or three times (Table S3.1).

Table S3.1. *The table shows the position allocation (role) for each participant for each round. The numbers in the table refer to a specific participant. For example, '1' stands for participant 1 who in round one was allocated the role of the Lead and in round two the role of the integrator, and so forth. Participants performed six trials in each round (position).*

Position	Lead	F1L	F2L	F1R	F2R	INT
Round 1	1	4	5	2	3	6
Round 2	6	5	4	3	2	1
Round 3	4	2	6	5	1	4
Round 4	2	3	1	4	6	5
Round 5	3	6	2	1	5	4
Round 6	5	1	3	6	4	2

3.2 Test for practice effect across positions

A repeated measures ANOVA was conducted for the variability of asynchrony and inter-movement-intervals (IMI), to test whether participants' performance improved across their positions. For both, the variability of IMI for fast tempo ($F(5,25)=0.980$, $p>.05$) and slow tempo ($F(5,25)=1.801$, $p>.05$; Fig. S3.1), no differences across positions were observed. Similarly, no differences were found for the variability of asynchrony for fast tempo ($F(5,25)=0.084$, $p>.05$) and slow tempo ($F(5,25)=0.636$, $p>.05$). Therefore, the experience of previous positions did not benefit the performance of a current position.

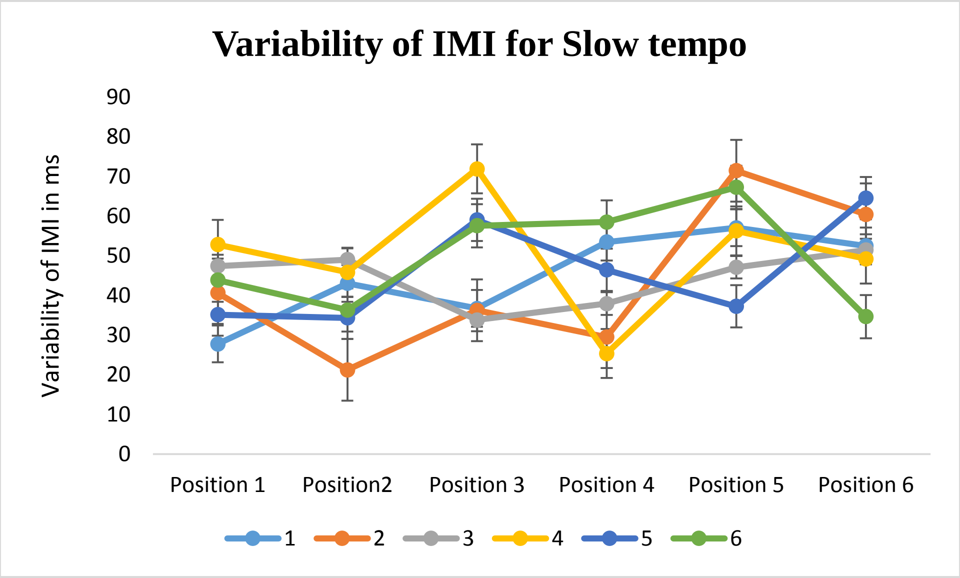

Figure S3.1. *Mean variabilities of IMIs for each participant's first up to their last (position number six) position are presented, for slow tempo only. Each line represents one participant.*

4. Supplementary Information S4

4.1 Mean alpha correction gains relative to all positions

We compared the mean alpha correction gain between adjacent position (e.g. LD position had the metronome as a cue) and all other positions (LF1, LF2, RF1, and RF2). As Figure S4.1 illustrates, the highest gain is always the adjacent position. Although we don't get zero gains for other positions due to the high levels of correlation within the group, all other gains are consistently lower. Note, we have left out the integrator as this position had two cues.

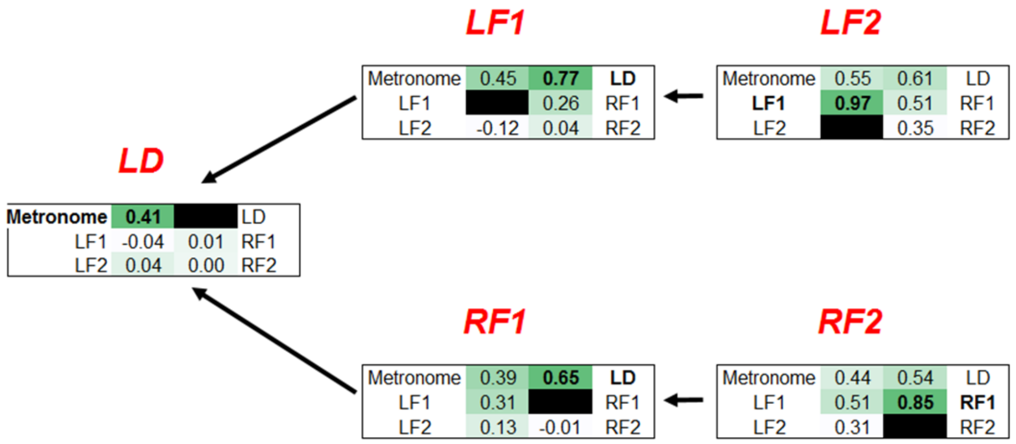

Figure S4.1. *Mean alpha gains relative to each other position for each role (e.g. RF1). The bold number and label shows the valid cue position (adjacent cue), and larger gains are highlighted with a darker green.*

References

1. Jacoby, N., Tishby, N., Repp, B. H., Ahissar, M. & Keller, P. E. Parameter Estimation of Linear Sensorimotor Synchronization Models: Phase Correction, Period Correction, and Ensemble Synchronization. *Timing Amp Time Percept.* **3,** 52–87 (2015).
2. Jacoby, N., Keller, P. E., Repp, B. H., Ahissar, M. & Tishby, N. Lower Bound on the Accuracy of Parameter Estimation Methods for Linear Sensorimotor Synchronization Models. *Timing Amp Time Percept.* **3,** 32–51 (2015).
3. Aitken, A. C. IV.—On Least Squares and Linear Combination of Observations. *Proc. R. Soc. Edinb.* **55,** 42–48 (1936).
4. Vorberg, D. & Wing, A. M. In Handbook of Perception and Action, Vol. 2 (eds Heuer, H. et al.) Ch.2, 181–262 (1996).
5. Vorberg, D. & Schulze, H. H. Linear Phase-Correction in Synchronization: Predictions, Parameter Estimation, and Simulations. *J. Math. Psychol.* **46,** 56–87 (2002).
6. Wing, A. M. Voluntary timing and brain function: an information processing approach. *Brain Cogn.* **48,** 7–30 (2002).
7. Vorberg, D. & Hambuch, R. Timing of Two-Handed Rhythmic Performancea. *Ann. N. Y. Acad. Sci.* **423,** 390–406 (1984).
